# miR‐455‐5p Overexpression Reduces Rat Lung Alveolar Type II Cell Proliferation by Downregulating *STRA6*

**DOI:** 10.1002/ar.24145

**Published:** 2019-06-02

**Authors:** Jintao Zheng, Qiuming He, Huajian Tang, Huimin Xia

**Affiliations:** ^1^ Department of Pediatric Surgery Foshan Maternity and Children's Healthcare Hospital Affiliated to Southern Medical University Guangzhou Guangdong China; ^2^ Department of Pediatric Surgery, Guangzhou Institute of Pediatrics, Guangzhou Women and Children's Medical Center Guangzhou Medical University Guangzhou Guangdong China

**Keywords:** miR‐455‐5p, *STRA6*, retinoid signaling pathway, lung development

## Abstract

miR‐455‐5p and retinoid signaling pathway and its membrane receptor, *STRA6*, are associated with lung development. Software copredictions indicate that the miRNA upstream of the *STRA6* gene is miR‐455‐5p. We hypothesized that miR‐455‐5p participates in rat lung alveolar Type II cell proliferation by targeting *STRA6* and designed this study to investigate the effects of miR‐455‐5p overexpression on rat lung alveolar Type II cells. Dual luciferase reporter gene assay was utilized to confirm the relationship between miR‐455‐5p and *STRA6*. An miR‐455‐5p‐expressing adenoviral vector was constructed and transfected into rat lung alveolar Type II cells. *STRA6* protein expression was detected in rat lung alveolar Type II cells by Western blotting at 72 hr posttransfection. Retinol concentration was detected by ELISA at 72 hr posttransfection. The cell proliferation was detected by CCK8 assay at 24, 48, and 72 hr posttransfection. Our results showed that *STRA6* is a target gene of miR‐455‐5p. *STRA6* protein expression was significantly lower in the miR‐455‐5p‐overexpression group than in the NC group (0.615 ± 0.131 vs. 0.958 ± 0.246, *P* = 0.029). Similar results were observed for retinol concentration (2.985 ± 0.061 vs. 3.949 ± 0.118, *P* = 0.000). Rat lung alveolar Type II cell proliferation was lower in the miR‐455‐5p‐overexpression group than in the NC group at 24, 48, and 72 hr posttransfection (24 hr: 0.280 ± 0.184 vs. 1.354 ± 0.169 *P* = 0.026; 48 hr: 0.881 ± 0.016 vs. 1.992 ± 0.050 *P* = 0.001; 72 hr: 2.105 ± 0.148 vs. 2.937 ± 0.079 *P* = 0.016). In summary, miR‐455‐5p is associated with lung development. miR‐455‐5p overexpression downregulates *STRA6*, leading to reduced retinol concentration and rat lung alveolar Type II cell proliferation. Anat Rec, 302:2062–2069, 2019. © 2019 The Authors. *The Anatomical Record* published by Wiley Periodicals, Inc. on behalf of American Association of Anatomists.

Fetal lung development is carefully orchestrated and precisely regulated by various mechanisms. Branching morphogenesis is driven by several carefully regulated processes, including cell growth and cell proliferation and differentiation. Branching morphogenesis is also regulated by apoptosis, which occurs in a space‐ and time‐dependent manner. Some of the signaling pathways involved in lung development have recently been discovered (Schnitzer, 2004). The retinoid signaling pathway is one of the most important signaling pathways in lung development (Tovar, 2012). Londhe et al. showed that retinoic acid (RA) rescues alveolar hypoplasia in the calorie‐restricted developing rat lung (Londhe et al., 2013), and Chen et al. provided evidence showing that prenatal retinoid deficiency leads to airway hyperresponsiveness in adult mice (Chen et al., 2014). Pulmonary dysplasia resulting from congenital diaphragmatic hernia (CDH) is one of the most common and important types of pulmonary dysplasia. Gene (*STRA6*, *RAR*, *FOG2*, *COUP‐TFII*, *GATA*), enzyme (RALDH) and protein (retinol binding protein; RBP) abnormalities in the retinoid signaling pathway led to pulmonary dysplasia in CDH in a nitrofen‐induced rat model (Mendelsohn et al., 1994; Huggins et al., 2001; al, 2003; Ackerman et al., 2005; You et al., 2005; Kutasy et al., 2012). Schmidt et al. discovered that antenatal treatment with RA reduced the median wall thicknesses of pulmonary arterioles in this model (Schmidt et al., 2016). Montedonico et al. demonstrated that RA rescues lung hypoplasia in nitrofen‐induced hypoplastic lungs by increasing the total DNA content, the proportions of proliferating cells, the numbers of lung buds, and the lung area (Montedonico et al., 2006). Some scholars were unable to obtain similar results in a series of studies using blood samples from children with CDH. However, some studies obtained similar results regarding *STRA6* expression. The molecular mechanism by which retinol is absorbed by cells from the retinol‐RBP complex was not completely understood until *STRA6* was identified as a membrane receptor specific for RBP in bovine retinal pigment epithelium cells (Kawaguchi et al., 2007). Pasutto et al. observed that mutations in *STRA6* lead to a variety of malformations, including lung hypoplasia, alveolar capillary dysplasia, diaphragmatic hernia, and congenital heart defects (Pasutto et al., 2007).We speculate that a key factor regulating *STRA6* gene expression in the retinoid signaling pathway causes pulmonary dysplasia in CDH. This key factor is likely to be an miRNA. miRNAs are small, noncoding RNA molecules comprising approximately 22 nucleotides that participate in mammalian oocyte growth and maturation, early embryonic development, stem cell lineage differentiation, and implantation through the posttranscriptional regulation of gene expression (Hossain et al., 2012). miRNAs play an important role in the regulation of lung development (Ambros, 2004). Dong et al. investigated the dynamic regulatory effects of miRNAs and found that miRNAs regulate target gene mRNA transcription levels independently, and thus affect target protein translation levels to regulate mouse lung organogenesis (Dong et al., 2010). Dicer is a crucial enzyme that converts miRNA and siRNA precursors into their short mature forms to regulate gene expression. Harris et al. inactivated Dicer in the mouse lung epithelium using a Dicer conditional allele and the sonic hedgehog (Shh) allele. Affected mice presented with lung dysplasia and respiratory failure after birth. These findings indicate that Dicer is essential for lung epithelial morphogenesis (Harris et al., 2006). Martinez‐AntonA discovered miR‐455 cut back and was involving in changes in mRNA for the epithelial cell marker. Transfection with miR‐455‐3p lead to changes in target protein expression. They thought changes in specific miRNAs during human airway epithelial cell differentiation control gene and protein expression important for differentiation (Martinez‐Anton et al., 2013). In summary, miRNAs and the retinoid signaling pathway are closely associated with lung development. Three software (TargetScan, MIRDB, http://microrna.org) copredictions indicate that the miRNA upstream of the *STRA6* gene is miR‐455‐5p. However, the association between miR‐455‐5p and the *STRA6* gene of the retinoid signaling pathway in lung development has not been studied. We designed this study to investigate this relationship.

## MATERIALS AND METHODS

### miRNA Prediction

We used three bioinformatics software programs (http://www.targetscan.org, http://mirdb.org, http://www.microrna.org) to reliably predict the upstream miRNAs of the *STRA6* gene and reduce the numbers of false positives and experimental errors.

### Construction of the pmiR‐RB‐REPORT Vector

The full‐length *STRA6* 3’UTR containing the putative miR‐455‐5p binding site was amplified from C6 genomic DNA by PCR and cloned into the pmiR‐RB‐REPORT vector (RiboBio Co., Ltd. Guangzhou, China) at the XhoI and NotI sites. The mutant *STRA6* vector contained two mutations. The primers used for this experiment were designed according to the *STRA6* 3’UTR sequences. The sequences of the primers specific for the wild‐type *STRA6* 3’UTR vector and the mutant *STRA6* 3’UTR vector were showed in Table 1. All the primers were synthesized by GENEWIZ Co., Ltd. (Suzhou, China). And the insertion of the wild‐type and mutant inserts was confirmed (Site 1: GGCACAT [40–46] mutated to CCGTGTA; and Site 2: GGCACAT [258–264] mutated to CCGTGTA) through sequencing by Guangzhou RiboBio Co., Ltd. (Guangzhou, China).

**Table 1:** The sequences of primers

rno_*STRA6*_F	5′‐GGCGGCTCGAGCCCTGCTTTTATGTGCTAA‐3′
rno_*STRA6*_R	5′‐AAT**GCGGCCGC**AGTGAGGAGGAGTGTTCCT‐3′
rno_*STRA6*_mut1	5′‐FGGCGG**CTCGAG**CCCTGCTTTTATGTGCTAA**CCGTGTA**TCTTGTCTACATCCTCCC‐3′
rno_*STRA6*_mut2_F	5′‐AAAGGGTG**CCGTGTA**TTAGGCCTCTCCTCCGCA‐3′
rno_*STRA6*_mut2_R	5′‐AGGCCTAA**TACACGG**CACCCTTTCCCTGACTGT − 3′
pre‐miR‐455‐5p F	5′‐ACGTGCGCTAGCGCTGGTTTAGTGAACCGTCAGATCCGCTAGCGGTGTGAGCGTATGTGCCTTTGGACTACATCGTGGACGCAGCACCATG‐3′
pre‐miR‐455‐5p R	5′‐TGCGTCGTGGTACGTCAGGTGCCCGTATATGTGAACGGAGTTCGAGCTCGAGTTCGAAGCTTAAGACGTCAGCTGCTCGAGGATTCC‐3′

### Luciferase Reporter Gene Assay

293 T cells in the logarithmic growth phase were cultured in a 96‐well plate at a density of approximately 1.5 × 10^4^ cells per well (a total volume of 100 μL/well) for 24 hr at 37°C with 5% CO_2_. Ten microliters of miR‐455‐5p mimics or nontarget controls (50 nM), 15 μL of pmiR‐RB‐REPORTvectors (250 ng/well), and 25 μL of Lipofectamine 2000 (0.25 μL/well) (Invitrogen) were mixed, gently shaken, and then incubated for 20 min. Fifty microliters of medium was removed from each well, and then 50 μL of the above mixture was added to each well. One hundred microliters of fresh medium were added to each well after 6 hr. The cells were collected at 48 hr after transfection and analyzed using a Dual‐Glo Luciferase Assay System (Promega). Luciferase activity was detected by Veritas 9100‐002 (Turner Biosystems), according to the manufacturer's protocol. The miR‐455‐5p mimic and nontarget control were synthesized by Guangzhou RiboBio Co., Ltd. The transfections were carried out in duplicate and were repeated independently at least three times.

### Construction of the miR‐455‐5p‐Overexpressing Adenoviral Vector

The pre‐miR‐455‐5p sequence was obtained from miRBase (http://www.mirbase.org) and was synthesized by GENEWIZ Co., Ltd. (Suzhou, China). The pre‐miR‐455‐5p sequence was amplified by PCR and cloned into the adenoviral vector PDC315‐EF1a‐egfp at the XheI and NotI sites. The sequences of the primers were showed in Table 1.

We confirmed that the sequence of the miRNA generated by the above experiment was identical to the known sequence of miR‐455‐5p through sequencing, which was performed by Guangzhou RiboBio Co., Ltd. (Guangzhou, China). The PDC315‐miR‐455‐5p‐EF1a‐eGFP vector and the coplasmid pBHGloxdelE13cre were mixed according to the Liposome transfection method, and then they were transfected into 293A cells for adenoviral amplification. The miR‐455‐5p‐overexpressing adenoviral vector was stored in liquid nitrogen after the viral titer was measured.

### Cell Culture and Adenovirus Transfection

**Figure 1:**
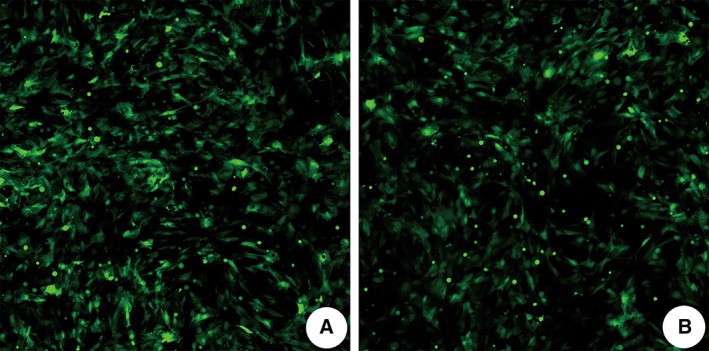
(**A**) Rat lung alveolar Type II cells in the miR‐455‐5p overexpression group transfected with overexpression adenovirus at 24 h posttransfection. (**B**) NC group. The transfection efficiency reached more than 90%, and the transfection effect was obvious.

Rat lung alveolar Type II cells (RLE‐6TN) were purchased from ATCC Co., Ltd. (ATCC Number: CRL‐2300) and cultured in DMEM (Gibco) at 37°C in a humidified atmosphere containing 5% CO_2_. All cell lines were authenticated and characterized by ATCC. The cells were divided into a miR‐455‐5p‐overexpression group and an NC group. Two hundred microliters of miR‐455‐5p‐overexpressing adenoviral vectors or NC adenoviral vectors were transfected into the cells in the miR‐455‐5p‐overexpression or NC groups, respectively (Fig. 1). Additional experiments were carried out at different time points.

### Western Blot Analysis

Total protein was extracted from the rat lung alveolar Type II cells after 48 hr of adenovirus transfection using Western Blot Box (Sigma‐Aldrich), and the protein concentration was measured using a BCA Protein Assay Kit (Thermo Fisher Scientific) according to the manufacturer's protocol. Ten microliters of protein were mixed with 40 μL of SDS‐PAGE loading buffer, after which the proteins were separated by SDS‐PAGE on a 10% gel and transferred onto nitrocellulose membranes, which were incubated with 5% skim milk powder for 2 hr at room temperature to prevent nonspecific binding before being incubated with the following primary antibodies overnight at 4°C: sheep antirat STRA6 (EVEREST BIOTECH, UK) and anti‐GAPDH (EVEREST BIOTECH, UK). The blots were subsequently incubated with horseradish peroxidase‐conjugated rabbit anti‐goat IgG secondary antibody (ThermoFisher Scientific). The signals were visualized by ECL. Changes in protein expression were quantified, and images were recorded using a BIO‐RAD Gel Doc XR+gel imaging system (BIO‐RAD).

### ELISA

Approximately 10^5^ cells were transferred to a cryopreserved tube at 48 hr after adenovirus transfection. One milliliter of PBS was added to the tube, after which the cells were frozen in liquid nitrogen and then placed in a water bath for 5 min 37°C. This procedure was repeated three times to induce cell lysis. A Rat Vitamin A ELISA Kit (Catalog Number: CSB‐E07890r) was used for this experiment and purchased from CUSABIO Co., Ltd., Wuhan, China. A standard curve was drawn using the standard sample in the kit according to the manufacturer's protocol. The absorbance of the cell sample was measured at a wavelength of 450 nm by a microplate reader. The relative concentration was calculated from the standard curve.

### Cell Proliferation

At 24 hr after adenovirus transfection, the rat lung alveolar Type II cells were recultured in a 96‐well plate (100 μL/well) and treated with 10 μL of Cell Counting Kit‐8 (CCK8) (Sigma‐Aldrich) reagent. The cells were then incubated in a humidified atmosphere of 5% carbon dioxide for 2 hr at 37°C. The absorbance of the cell sample was measured at a wavelength of 450 nm using a microplate reader. The same experiment was repeated at 48 and 72 hr posttransfection.

## RESULTS

### miR‐455‐5p Regulates *STRA6* Expression

**Figure 2:**
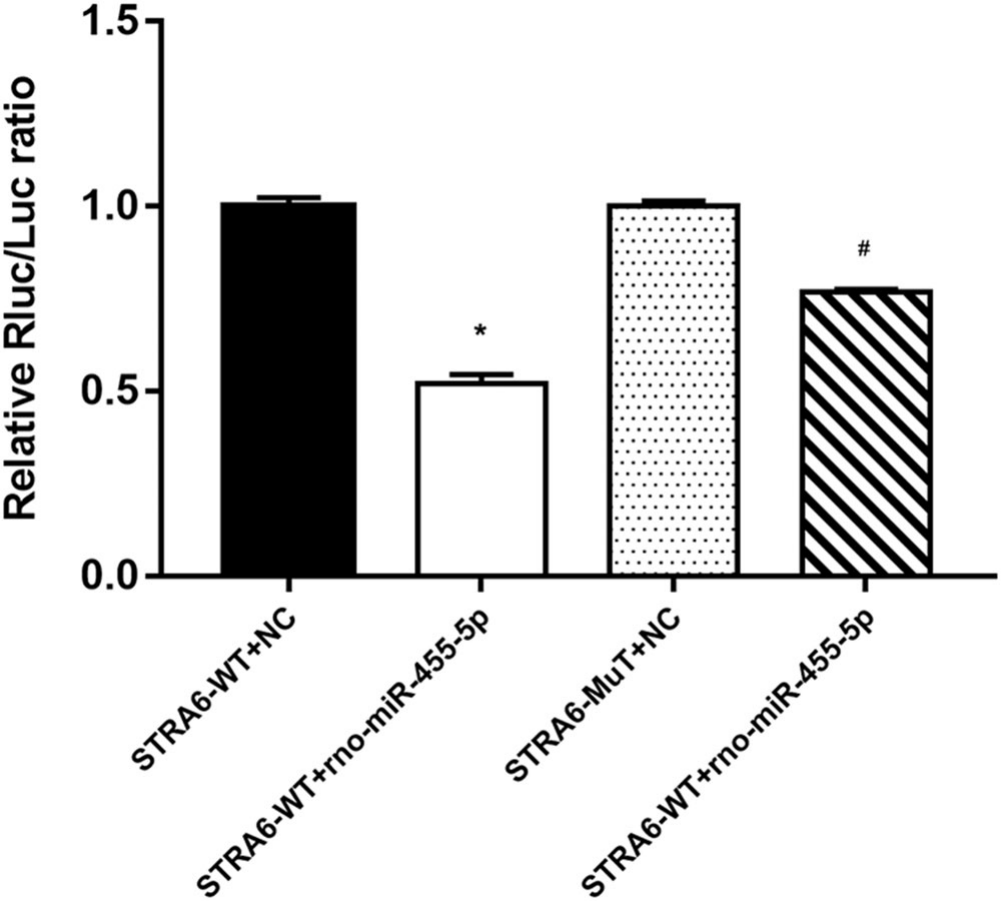
Dual luciferase reporter assay results (relative fluorescence values) for each group. Transfection with the rno‐miR‐455‐5p mimic significantly downregulated the reporter fluorescence of wild‐type STRA6 (47.9%) compared with transfection with the NC. Mutating the predicted target site improved the reporter fluorescence. However, the fluorescence level (23.2%) in miRNA mimic‐transfected cells was still lower than that in NC‐transfected cells. “*” and “#” indicated *P* < 0.05 between STRA6‐WT + NC and STRA6‐WT + rno‐miR‐455‐5p and between STRA6‐Mut + NC and STRA6‐Mut + rno‐miR‐455‐5p, respectively.

The analysis of dual luciferase reporter gene expression showed that under normal conditions, miRNA mimics reduce fluorescence by at least 30% compared with NCs. This finding suggests that miRNA mimics have regulatory effects on the reporter gene. The rno‐miR‐455‐5p mimic significantly downregulated the reporter fluorescence of wild‐type *STRA6* (47.9%) compared with the NC. Mutating the predicted target site of miR‐455‐5p improved the reporter fluorescence. However, the fluorescence level in miRNA mimic‐transfected cells (23.2%) was still lower than that in NC‐transfected cells. This result indicates that rno‐miR‐455‐5p regulates *STRA6* gene expression by binding to its specific target site in the *STRA6* 3’UTR (Fig. 2).

### miR‐455‐5p Overexpression Reduced *STRA6* Protein Expression

**Figure 3:**
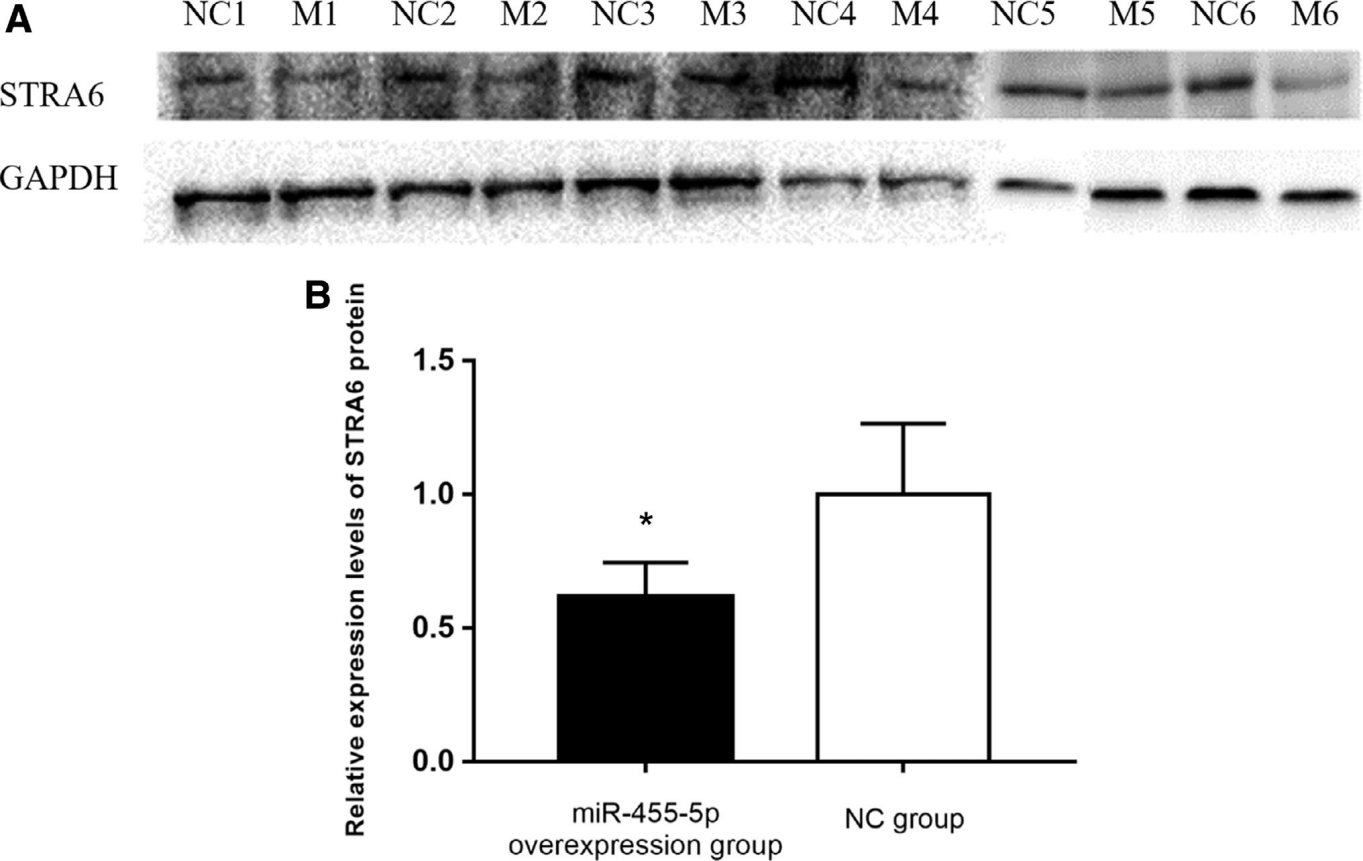
(**A**) Western blotting analysis of STRA6 protein expression. NC indicates the NC group, and M indicates the miR‐455‐5p‐overexpression group (*n* = 6). GAPDH was used as an internal reference. (**B**) The relative expression level of STRA6 protein in the miR‐455‐5p‐overexpression group was significantly decreased compared with that in the NC group, “*” means *P* < 0.05.

Western blot analysis showed that the relative expression of *STRA6* protein was significantly downregulated after transfection with the miR‐455‐5p‐overexpressing adenoviral vector for 48 hr compared with transfection with the NC adenoviral vector (*P* < 0.05) (Fig. 3). These results showed that miR‐455‐5p overexpression downregulates *STRA6* protein expression in rat lung alveolar Type II cells.

### miR‐455‐5p Overexpression Diminished Cellular Retinol Concentration

**Figure 4:**
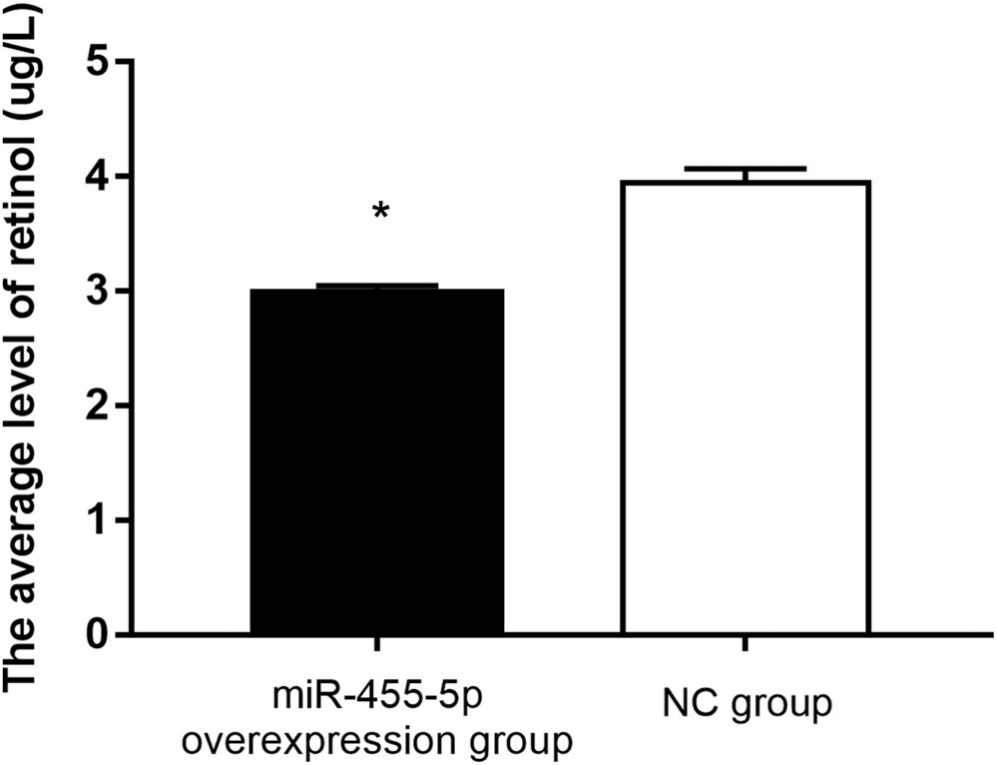
The retinol level the in the miR‐455‐5p‐overexpression group was significantly decreased compared with that in the NC group. “*” means *P* < 0.05.

The average retinol level was calculated by a standard curve. The retinol level in the miR‐455‐5p‐overexpression group was significantly decreased compared with that in the NC group (*P* < 0.05). These results show that the retinol level was decreased after miR‐455‐5p was overexpressed in rat lung alveolar Type II cells (Fig. 4).

### miR‐455‐5p Overexpression Influenced Rat Lung Alveolar Type II Cell Proliferation

**Figure 5:**
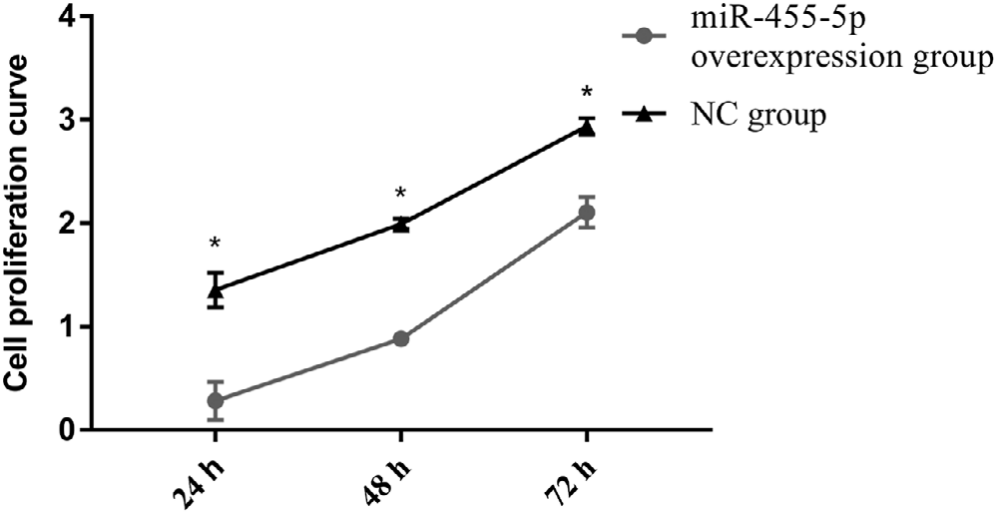
The data in the above graph show that the rat lung alveolar Type II cell proliferation rate in the miR‐455‐5p‐overexpression group was significantly decreased compared with that in the NC group at 24, 48, and 72 h posttransfection. Cell growth slowed significantly in the miR‐455‐5p‐overexpression group after 72 h posttransfection. “*” means *P* < 0.05 between miR‐455‐5p‐overexpression and NC groups.

The rat lung alveolar Type II cell proliferation rate in the miR‐455‐5p‐overexpression group was significantly lower than that in the NC group at all three time points (*P* < 0.05). The cell proliferation rate increased significantly in both groups (*P* < 0.05). However, the cell proliferation rate was significantly reduced in the miR‐455‐5p‐overexpression group compared with the NC group (Fig. 5).

## DISCUSSION

Retinol and its active metabolites (RA) are essential for lung development. The maintenance of a proper retinol level in the embryo is important. Retinol metabolic abnormalities may lead to a variety of congenital malformations, including malformations affecting the respiratory system (Zile, 2001; Clagett‐Dame and DeLuca, 2002). James et al. established a hyperoxia‐induced lung injury model in C57BL/6 mice, which they treated with a VitA‐RA mixture. They found that the mixture enhanced lung retinol stores and lung compliance while weakening hyperoxia‐induced alveolar simplification. Therefore, they concluded that the VitA‐RA mixture is a means of treating VitA deficiency‐ and hyperoxia‐induced lung injury during lung development (James et al., 2013). Fraslon et al. administered a single 50,000‐IU dose of retinyl palmitate to pregnant rats on day 16 of pregnancy, and then they detected total phospholipid (TPL) and desaturated phosphatidylcholine (PC) levels in fetal surfactant on day 19. They discovered that TPL and PC levels increased by 22% and 29%, respectively (Fraslon and Bourbon, 1994). Therefore, they concluded that fetal lung surfactant phospholipid levels were increased by chronic RA administration. Meyer et al. carried out a multicenter double‐blinded RCT in which they administered high‐dose oral vitamin A (5,000 IU vitamin A/kg/day) or placebo to extremely low birth weight infants for 28 days to determine whether VitA reduces bronchopulmonary dysplasia (BPD) or mortality rates compared with placebo. All the infants required mechanical ventilation, noninvasive ventilatory support, or supplemental oxygen beginning at 24 hr of age. The RCT provided robust data indicating that high‐dose oral VitA supplementation prevents BPD and death (Meyer et al., 2014).

The retinoid signaling pathway comprises a variety of genes and factors, and the mechanisms by which the pathway is regulated are relatively clear (Noble et al., 2007). *STRA6* belongs to a family of proteins stimulated by RA and is a specific membrane receptor for RBP. STRA6 recognizes the VitA–RBP complex with high affinity and transports VitA between extra‐ and intracellular RBP (Kawaguchi et al., 2007; Kelly and von Lintig, 2015). *STRA6* protein expression directly affects retinol absorption, and thus affects intracellular retinoid signaling pathway activity.

Multiple clinical syndromes characterized by pulmonary hypoplasia have been identified. Some of these syndromes are associated with genetic variations in *STRA6*. PDAC syndrome is characterized by pulmonary hypoplasia, diaphragmatic hernia, anophthalmia, and cardiac malformations and is related to recessive mutations in the *STRA6* gene. Segel et al. described patients who were compound heterozygotes for two novel *STRA6* missense mutations associated with PDAC syndrome (Segel et al., 2009). Chassaing et al. identified two patients with *STRA6* gene mutations who had some or all of the features of PDAC syndrome. One patient was a compound heterozygote for a missense mutation and a large intragenic deletion, and the other patient was homozygous for a splicing mutation (Chassaing et al., 2013). Golzio et al. analyzed the *STRA6* genes of two human fetuses with Matthew‐Wood syndrome from consanguineous families and found that the fetuses had a homozygous insertion/deletion in exon 2 and a homozygous insertion in exon 7, respectively. Matthew‐Wood syndrome is characterized by pulmonary agenesis, severe microphthalmia, intrauterine growth restriction, and bilateral diaphragmatic eventration (Golzio et al., 2007).

miRNAs are small noncoding RNAs comprising 22 nucleotides that regulate gene expression at the posttranscriptional level by targeting mRNAs for cleavage or translational repression. miRNAs are known to play an important role in lung development (Bartel, 2004). Mice lacking the miR‐17‐92 cluster died shortly after birth because of lung hypoplasia and a ventricular septal defect (Ventura et al., 2008). Overexpression of the miR‐17‐92 cluster in the lung epithelium promoted cellular proliferation and inhibited differentiation (Lu et al., 2007), while targeted deletion of the miR‐17‐92 and miR‐106b‐25 clusters resulted in embryonic or early postnatal lethality (Carraro et al., 2009). miR‐449a is highly expressed specifically in pulmonary tissue and may actively promote mucociliary differentiation (Lize et al., 2010). Wang and colleagues found that miR‐375 is downregulated during lung alveolar epithelial cell differentiation. Alveolar epithelial cell differentiation was restrained after the transfection of a miR‐375‐overexpressing adenoviral vector (Wang et al., 2013). Tian and colleagues showed that miR‐302 and 367 affect progenitor cell proliferation, differentiation, and apical‐basal polarity, and thus regulate lung endoderm development by targeting the transcription factor *Gata6* (Tian et al., 2011). Shh is highly expressed in the mouse embryonic lung epithelium and is essential for lung development. Jiang et al. demonstrated that miR‐326 regulates the Shh signaling pathway through a negative feedback mechanism by directly targeting the *Smo* and *Gli2* genes, thereby affecting early lung development (Jiang et al., 2014). Normal miR‐125b and miR‐30a/c expression can suitably regulate Snail1 gene expression, which is necessary for normal trachea development. Gradus et al. enhanced Snail1 gene expression by limiting miR‐125b and miR‐30a/c expression in chondrocytes or knocking out Dicer1 in the trachea. These authors showed that Aggrecan and Col2a1 transcription is inhibited, and extracellular matrix deposition is reduced as a result of the above changes (Gradus et al., 2011). miRNAs are generally believed to play a critical functional role during embryonic lung development.

**Figure 6:**
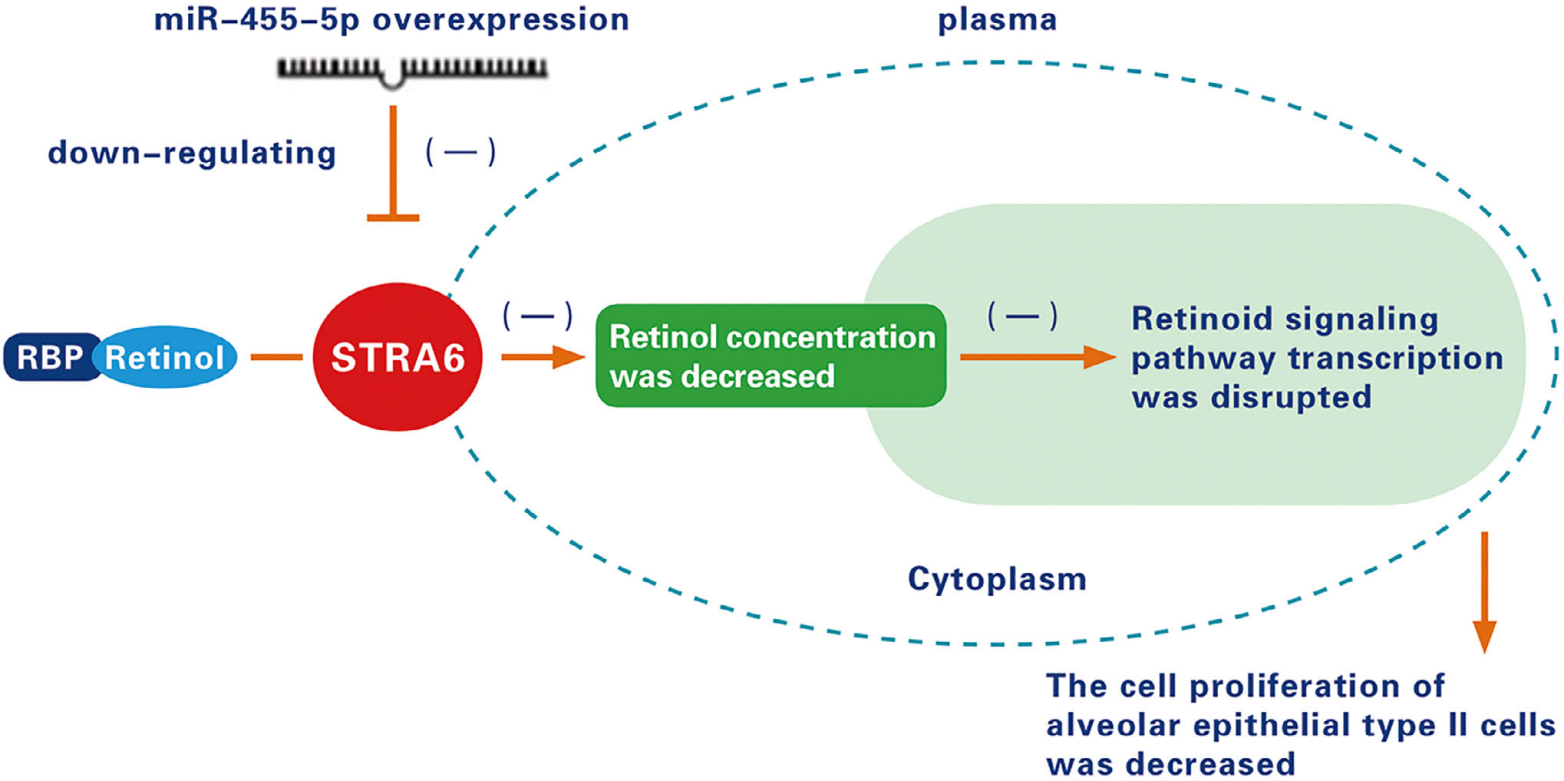
miR‐455‐5p reduces rat lung alveolar Type II cell proliferation by down‐regulating STRA6 of the retinoid signaling pathway.

In summary, miRNA and the retinoid signaling pathway and its membrane receptor, STRA6, are associated with lung development. In this study, we succeeded in determining the relationship between miR‐455‐5p and *STRA6* (Fig. 6). First, we used biological software to predict the miRNA upstream of STRA6. We found that miR‐455‐5p was the miRNA upstream of *STRA6*. This result was confirmed by dual luciferase reporter gene assay. Second, we constructed a miR‐455‐5p‐overexpressing adenoviral vector and transfected it into rat lung alveolar Type II cells for several time periods. Western blotting showed that miR‐455‐5p downregulates *STRA6* protein expression, and ELISA showed that miR‐455‐5p significantly reduces retinol concentration. CCK8 assay showed that rat lung alveolar Type II cell proliferation was significantly decreased after retinoid signaling pathway transcription was disrupted.

In conclusion, miR‐455‐5p is associated with lung development. miR‐455‐5p overexpression downregulates STRA6 that may lead to reduced retinol concentration and rat lung alveolar Type II cell proliferation. But the results have a limitation, because the experiment was carried out *in vitro*, using lung alveolar Type II cells that are immortalized (RLE‐6TN). A next step will be to test the outcomes of the present study by assessing whole lung *in vitro* or doing animal experiments.
